# Performance Evaluation of Out-of-Spec Carbon Prepregs for Upcycling Purposes

**DOI:** 10.3390/polym16121625

**Published:** 2024-06-07

**Authors:** Sanjeev Rao, Dereck Bastienne

**Affiliations:** Department of Aerospace Engineering, Khalifa University of Science and Technology, Abu Dhabi P.O. Box 127788, United Arab Emirates; 100035546@kustar.ac.ae

**Keywords:** automated fiber placement, upcycling, out-of-spec prepregs, cure kinetics, DMA

## Abstract

In view of exploring the possibility of upcycling aerospace scrap, cure characteristics of out-of-spec carbon fiber prepregs are investigated in this study. The cure behavior of the prepreg is examined in the form of the mechanical cure conversion state of the material using a Dynamic Mechanical Analyzer (DMA). Cure kinetics is modeled by comparing the storage modulus at the start of the reaction (E′_0_) and instantaneously (E′_t_) during isothermal experiments with those of the fully cured material (E′_∞_) obtained from dynamic scans. The glass transition temperature T_g_ and the extent of reaction before gelation are modeled using the DiBenedetto model, where the T_g_ of each laminate is determined in a DMA, per standard ASTM D7028. The mechanical properties, the extent of cure, and the glass transition temperature of the cured laminates were determined according to industry and international standards. The maximum conversion at temperatures between 100 °C and 140 °C is approximately 80% (±5%). The modeled rate of conversion shows a reasonable match with the experimental data, exhibiting a maximum reaction rate at about 30–40% of the cure conversion. The predicted evolution of the T_g_ as a function of cure conversion using the DiBenedetto model provides a 94% match with the experimental data. The multi-stage cure cycle based on the models offers shorter cycle times and high-quality laminates. The mechanical test results indicate approximately a 13% and 15% decrease in tensile strength and modulus, respectively, compared to pristine ones. The experimental extent of cure of the cured laminates (95.4%) is in close agreement with that predicted by the model (97%). The porosity in the laminates is estimated to be approximately 2.4%, which is acceptable in several industries.

## 1. Introduction

The Automated Fiber Placement (AFP) technology evolving from the Automated Tape Laying (ATL) process has significantly advanced the aerospace manufacturing sector. Since its introduction in the 1980s, it has been widely implemented across several aerospace industries. The process uses narrow slit tapes in the range of ¼ to ½ inch width compared to the 6-to-11-inch tapes used in the conventional tape laying process. The narrow width of the tape provides the user control over steering angles during placement, reduces fiber buckling associated with conventional ATL methods, and reduces material wastage. The AFP provides on-the-fly material debulking due to in situ compaction from the rollers and application-based cutting alternatives, which are a few of the advantages of the process over the traditional ATL method. This progression in technology also led to the development of Out-of-Autoclave (OoA) slit tapes, presenting a cost-efficient alternative to conventional autoclave processing, and expanding the possibilities for material application. The oven curing process is a two-step process based on a primary low-temperature vacuum bag curing and a high-temperature free-standing post-cure to obtain a high glass transition temperature (T_g_) of the laminate. Given that autoclaves are not involved, the technology drastically reduces the initial investment of the process and can be easily adapted to a variety of components’ shapes and sizes, resulting in a much more versatile methodology. However, irrespective of the advantages the process has to offer, there is some form of material wastage arising from offcuts from ply-cutting operations, skeletons, trim waste, end-of-roll waste, and out-of-spec material (within the aerospace industry, defined as the materials surpassing their expiration date as provided by the manufacturer). It is cost-prohibitive in the aerospace industry to re-certify such out-of-spec material, and it is, therefore, disposed of in landfills, donated to research institutes, or consumed in-house for research purposes.

In recent times, legislative changes, particularly the End-of-life Vehicle Directive (2000/53/EC) [1], have significantly propelled the focus toward the recycling and upcycling of materials, emphasizing the need for sustainable practices. Recent advancements in the recycling of carbon fiber composites, especially through pyrolysis and solvolysis, have shown promise in reclaiming fibers with minimal loss of integrity, aligning with the goals of environmental sustainability [2,3]. Recycling carbon fiber composites provides great potential in reducing both cost and environmental effects and can generally be categorized into three main groups: mechanical, thermal, and chemical. In mechanical recycling, composite material waste is shredded between blades in mechanical shredders, and the resulting pieces or powder form are used as fillers in other applications, such as roads, concrete bricks, etc. This process is mostly effective for fiber glass parts that have been manufactured using bulk molding or sheet molding compounds, and reclaiming fibers using expansive and expensive processes may not be commercially or environmentally beneficial [4]. A popular and effective process where expensive carbon fibers can be reclaimed is pyrolysis, a thermal recycling process where the resin portion of the composite is eliminated via decomposition in a high-temperature furnace with an inert atmosphere. In recent times, this process has received substantial attention because of the possibility of reclaiming fibers that may possess properties near their virgin counterparts. For example, in a recent work by Abdou et al. [5], the authors have reported that it is possible to obtain fiber surface morphologies close to that of the virgin fibers when the pyrolysis is conducted at 550 °C for a period of 1 h. Yang et al. [6] have shown that it is possible to retain approximately 80% of fiber tensile strength if the composite material was processed at 650 °C in an atmosphere consisting of 5% oxygen for a period of 45 min. Kim and co-workers [7] have been able to retain 90% of the tensile strength by exposing carbon fiber-reinforced composites to superheated water steam at 550 °C for 30 min, followed by oxidation in midair at 550 °C for 30–75 min. Jiang et al. [8] conducted pyrolysis in a nitrogen-purged microwave furnace at various temperatures of 400, 500, and 600 °C for 30 min and revealed that it was not possible to obtain completely clean fibers, even at high temperatures. Furthermore, Wei and S.A. Hadigheh [9] described the low-temperature pyrolysis combined with solvolysis pre-treatment as an effective recycling method for carbon fiber-reinforced plastic (CFRP) waste. Stefania Termine et al. [10] reported that the pyrolysis process can reclaim carbon fiber fabrics for reuse, and post-pyrolysis treatment improves fiber wettability but decreases mechanical properties. Gabriel Nicolai [11] illustrated that thermochemical recycling is an effective way to reuse carbon fiber, and recycled materials preserved physical structure as well as tensile strength. In another work, Wong and co-workers [12] reported that conventional pyrolysis techniques are sufficient to yield fibers that can be comparable to their virgin counterparts, and other elaborate methods using mechanical, combustion, gasification, or slow pyrolysis techniques are costly and may indeed yield poor-quality fibers. Although thermal recycling methods may yield fibers with properties closer to virgin ones, the recycling process, especially pyrolysis, results in fibers with considerable char formation on the surface. Another efficient way is chemical recycling, where the matrix material is separated from the fibers using solvolysis, glycolysis, and hydrolysis techniques [13,14,15,16]. The process is able to extract fibers with no surface char and negligible loss in their mechanical properties. Researchers [17,18,19] have worked on the chemical recycling process and have reported the properties of the recovered fibers to be at least 80% compared to virgin ones. Whilst several researchers have focused on recycling and methods to recover and reclaim reinforcing fibers for reuse, few have paid attention to upcycling carbon scrap that is generated during production. The process involves reusing scrap prepreg materials resulting from ply cutter waste, end-of-roll, or expired material to manufacture new composite parts. Nilakantan and Nutt [20] have presented a comprehensive study on a practical and scalable way to process carbon fiber/epoxy prepreg scrap. The resulting material can then be readily used to manufacture new products using hot pressing and compression molding techniques. Kiss and co-workers [21] have used tattered layers between fiber films to produce compression-molded plates that exhibit impact properties as high as 50% of those manufactured using virgin materials. Several research [20,22,23] regarding upcycling focused on the mechanical properties of the byproduct, and few have focused on the characterization of the scrap in order to use them efficiently. Therefore, in view of exploring the viability of upcycling out-of-spec prepreg materials to manufacture sports equipment, investigations have been carried out in this work to understand the change in cure characteristics of the out-of-spec material. This work differs from those existing in the literature by presenting the bottom-up approach to productively upcycle prepreg scrap. The cure kinetics of the out-of-spec prepreg material is modeled based on the mechanical cure conversion state of the material via a Dynamic Mechanical Analyzer (DMA), in essence allowing the evaluation of the mechanical properties of the entire fiber-resin system.

## 2. Materials and Methods

An Out-of-Autoclave (OoA) towpreg, CYCOM^®^ 5320-1, supplied by Cytec/Solvay Engineered Materials Inc., Woodland Park, NJ, USA was used in this study. The OoA slit tape is specifically designed for the Automated Fiber Placement (AFP) process, consisting of IM7 carbon fibers impregnated in toughened CYCOM^®^ 5320 epoxy resin. The material had surpassed its shelf life by a year but was stored in refrigerated conditions during that period of time.

The mechanical cure characteristics of the out-of-spec towpreg were investigated via experiments in a DMA 8000 equipment from Perkin Elmer^®^, Waltham, MA, USA. Isothermal and dynamic scan experiments were carried out at a frequency of 1 Hz under strain control mode, using a single cantilever setup [24,25]. Isothermal experiments were conducted at 10 °C intervals between 100 °C and 140 °C, and dynamic scans were carried out at 2 °C/min between −30 °C and 300 °C. The dry glass transition temperature ‘T_g_ dry’ was determined as per standard AITM-0003 [26] on cured samples that were prepared in accordance with standard EN 2565 [27]. Five samples were tested in total, and the average T_g_ dry was determined using tangents across loss moduli traces and tan δ peaks.

**Figure 1:**
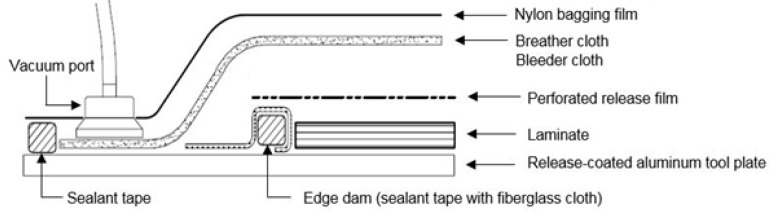
Laminate bagging setup sequence.

Simple balanced symmetric laminates [90/+45/0/−45/90] were manufactured using an in-house fiber placement system that consisted of a roller and a 20 kN load cell (Omegadyne LCM202) housed between the roller and the tool arbor of a Bridgeport^®^ milling machine. The layup temperature was monitored using an infrared camera (FLIR A655sc), and a hot air gun from Bosch^®^, Abu Dhabi, United Arab Emirates, was used as the heat source [24]. The obtained thin laminates (<2 mm) were cured using only a vacuum bag in a convection oven. The two-step curing cycle with two ramps and two holds at 121 °C and 177 °C was designed based on mechanical curing experiments using DMA techniques. The multi-stage cure cycle consisted of a temperature ramp from ambient until approximately 120 (±5) °C at the rate of 2 °C/min, followed by a temperature isotherm dwell stage for approximately 1 h, during which edge devolatilization and temperature uniformity between the tooling and laminate is attained. An aluminum flat plate tooling of 5 mm was used to cure all the plies, and it was determined via experimentation in a convection oven that 2 °C/min provided uniform heating throughout the plate. The layup and consumables used in this study, shown in Figure 1, consisted of an edge dam with 80 g/m^2^ plain weave glass for edge breathing/bleeding and a perforated release film over which a 140 g/m^2^ polyester bleeder/breather (N4 from Airtech^®^, Huntington Beach, CA, USA) was used for face bleeding. High-temperature vacuum tape AT-200Y from Airtech^®^ was used to seal the nylon vacuum bags (Airtech^®^, Wrightlon^®^ 7400). Further details of the hardware set-up and the processing conditions for the automated layup can be found in [28].

Tensile tests were conducted on manufactured coupons as per standard ASTM C 3039 using an Instron universal testing machine with a 50 kN load cell. The calibration results of prior experimentations showed a maximum error at 125 N to be 0.05% (0.0625 N) with a repeatability of 0.05%. An extensometer with a gauge length of 50 mm was used to record the strain. The voids and porosities were determined using a Matlab^®^ (R2023a) program from 2D optical images of the sample cross-sections using a Zeiss AX10 stereo microscope.

The measurement of the degree of cure α′ was established in accordance with the prescribed AITM 3-0008 standard [26], employing the Perkin Elmer DSC 8500 instrument subsequent to the conditioning of the specimens, following the guidelines outlined in EN2743 [29]. Determination of the degree of cure was carried out via the utilization of Equation (1), with an accompanying adjustment for 100% resin content achieved through the implementation of EN 2559 [30], thereby facilitating the determination of the authentic resin content present within the system.

(1) $$\alpha^{\prime }=\frac{∆H_{A}-∆H_{B}}{∆H_{A}}100[\%]$$

where $∆H_{A}$ is the reaction of enthalpy in Joules of the A curve, which is heated at a rate of 10 °C/min, and $∆H_{B}$ is the reaction of enthalpy in Joules of the B curve, which is subjected to the curing cycle that is being investigated.

Thermal scans were conducted for the uncured prepreg from either ambient or sub-ambient (−40 °C) up to 300 °C for a series of ramp rates: 1 °C/min, 1.5 °C/min, and 2 °C/min. It is a quick method used to study thermal transitions and also provides estimates for the glass transition temperature of the uncured and fully cured material. Isothermal curing experiments were conducted within a temperature range of 100 °C to 140 °C, with a 10 °C increment, in order to investigate the cure progression as a function of time and temperature.

Due to thermal overshoots resulting from the swift temperature rise, the temperature was allowed to stabilize within ±2 °C of the target isotherm, which took about 14 to 20 min after the starting time, depending on the temperature. For each isothermal temperature, the test was made to run up until the reaction was effectively quenched, that is, when the rate of increase in storage modulus tends to zero, which is analogous to the heat flow approaching zero in DSC experiments.

### 2.1. Cure Kinetics Modeling

The cure conversion of the towpregs was calculated using Equation (2) [24]. For each curing temperature, the storage modulus at the beginning of the reaction (${E^{\prime }}_{0}$) and the instantaneous storage modulus at different times (${E^{\prime }}_{t}$) were obtained from the isothermal tests, while the maximum storage modulus value of the fully cured material (E′_∞_) was acquired from the dynamic scan.

(2) $$\alpha =\frac{{E^{\prime }}_{t}-{E^{\prime }}_{0}}{{E^{\prime }}_{\infty }-{E^{\prime }}_{0}}$$

The rate of reaction (dα/dt) was obtained by differentiating the cure conversion curves with respect to time. Within the isothermal processing conditions considered, a good correlation between the model and the experimental data was obtained using the modified autocatalytic kinetic model developed by Kamal and Sourour [31], which accounts for the diffusion reaction control due to vitrification, as outlined below.

(3) $$\frac{d\alpha }{dt}=\left(k_{1}+k_{2}\alpha^{m}\right){\left(1-\alpha \right)}^{n}$$

where α is the cure conversion and m and n are the reaction orders. k_1_ and k_2_ are the reaction constants and follow the Arrhenius temperature-dependent relationship given by

(4) $$k_{i}=k_{0i}\exp\left(\frac{-E_{ai}}{RT}\right)\;for\;i=1,2$$

where k_0_ is the pre-exponential factor, E_a_ is the apparent activation energy, R is the gas constant, and T is the absolute temperature.

### 2.2. Gel Time and Vitrification Time Evaluation and Modeling

Gelation was estimated to occur at the inflection point of the storage modulus curve during the isothermal curing. The theoretical gel time as a function of cure kinetics is expressed in Equation (5) [32]

(5) $$t_{gel}=\frac{1}{K(T)}\int_{0}^{\alpha_{gel}}\frac{1}{f(\alpha )}d\alpha$$

where $K\left(T\right)$ is the kinetic parameter and f(α) is the function of the reaction mechanism and cure conversion. Flory’s gelation theory [33] can then be used to linearize it (Equation (6)). The apparent activation energy (E_a_) is obtained from the slope of Equation (6).

(6) $$\ln\left(t_{gel}\right)=\frac{E_{a}}{RT}+constant$$

Vitrification during curing appears when the glass transition temperature of the crosslinked polymer reaches the curing temperature. In this experiment, the start of vitrification on the storage modulus curve is representative of E′, attaining a maximum value asymptotically.

### 2.3. DiBenedetto Model

Several laminates were cured at 120 °C for various periods of time in order to reach a pre-defined fractional conversion (x) estimated from the cure kinetics model. Subsequently, the T_g_ of each laminate was determined by a dynamic DMA scan, in accordance with ASTM D7028 [34]. Since T_g_ is sensitive to the overall dimension of the sample, each test sample was one layer thick in order to minimize random errors when it comes to the computation of the T_g_ values. The onset of the sharp drop in the storage modulus was used as the criterion for determining the glass transition temperature. Three samples were tested for each degree of cure, and an average was taken. The variation of the T_g_ with respect to the cure conversion was modeled with the DiBenedetto equation [35] as follows:

(7) $$T_{g}=T_{g0}+\frac{\lambda x(T_{g\infty }-T_{g0})}{1-(1-\lambda )x}$$

where $T_{g0}$ and $T_{g\infty }$ are the glass transition temperatures of the unreacted and the fully cured system, respectively. λ is obtained by fitting Equation (7) to experimental data of the degree of cure and glass transition temperature, which is a limiting case; when α = 0, then $T_{g}=T_{g0}$, and when α = 1, $T_{g}=T_{g\infty }$.

## 3. Results and Discussion

### 3.1. Cure Kinetics

**Figure 2:**
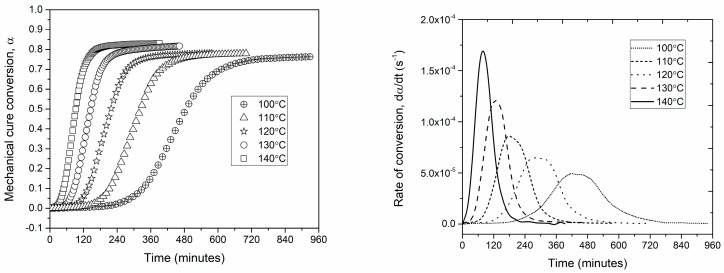
Time evolution of the mechanical cure at various isothermal temperatures (**left**) and time-evolution of the conversion rate at various isothermal temperatures (**right**).

The cure kinetics of the Out-of-Autoclave (OoA) towpreg investigated via DMA analysis in this work enables the evaluation of the mechanical properties of the entire fiber-resin system during the curing process. The instantaneous cure conversion (α) as a function of time was determined as per Equation (2) using a series of isothermal curing traces. In Figure 2, the conversion is characterized by three stages. The first one is a flat region, indicating negligible cure that monotonically decreases with the increase in testing temperatures; the second one is a rapid increase in conversion due to the change in the rate of the reaction until it asymptotically attains a maximum value, which is followed by the third region, where the rate of reaction sufficiently slows down to exhibit a plateau. This is caused because of the crosslinking-induced vitrification, whereby the molecular mobility is hindered, and the reaction assumes diffusion-controlled kinetics. The time evolution of the reaction rate for the series of isothermal cure processes (Figure 2) was obtained by differentiating the cure conversion curves with respect to time. All patterns are well described by Gaussian functions, and a monotonic increase in the reaction rate with the curing temperature is verified.

The key observation in Figure 2 is that the rate of the reaction progresses with the increase in temperature, confirming the ability of the system to achieve a cure. The cure conversion percentage is an estimate of how much resin has already been cured at that temperature, and the information is required to apply autoclave pressure such that the voids and porosities are squeezed out of the laminate. A higher conversion rate means higher resin viscosity, and the propensity of voids and trapped porosities in the laminate is high, and at a lower conversion, the resin viscosity is low, and the application of pressure may lead to resin bleeding and dry spots.

**Figure 3:**
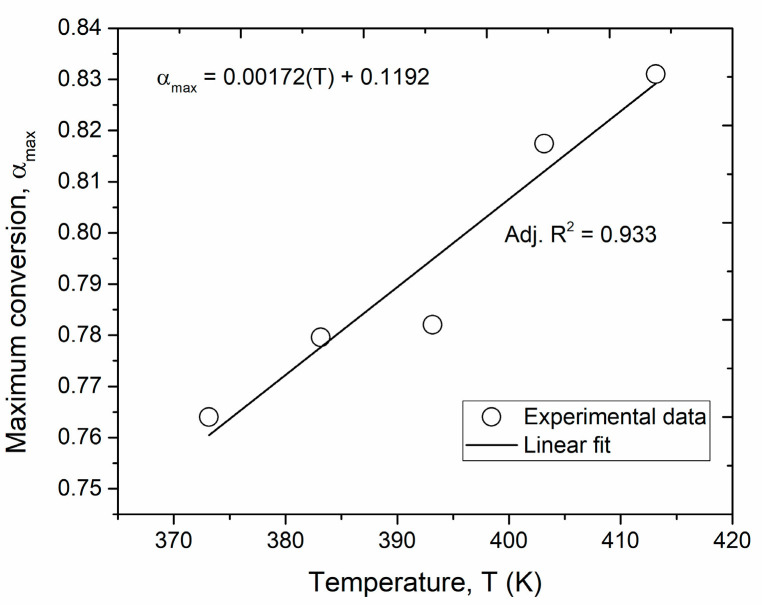
Maximum conversion as a function of the isothermal curing temperature.

The maximum conversion at temperatures between 100 °C and 140 °C is approximately 80% (±5%). The modeled rate of conversion plotted as a function of the mechanical cure conversion in Figure 3 shows a reasonable match with the experimental data, exhibiting the maximum reaction rate at about 30–40% of the cure conversion. The maximum fractional conversion ($\alpha_{max}$) was found to be dependent on the cure temperature and is expressed as a linear relationship based on the experimental data (Figure 3). Furthermore, the obtained values for the maximum achievable conversion at each temperature are in agreement with those previously reported in the literature [36,37,38].

The constants and exponents of the autocatalytic model ($k_{1}$, $k_{2}$, m, and n) were determined using a non-linear least-square fit, based on the Levenberg Marquardt algorithm, for each isothermal ‘rate of reaction’ curve. The total reaction order (m+n) was found to be relatively insensitive to the cure temperature, resulting in an average value of approximately 2. This is in close agreement with several thermoset polymerization reactions found in the literature [39,40], further validating the assumption of the cure mechanism to remain unchanged in the temperature band considered in this study. The reaction constants and the activation energies were calculated from the linear region of ln⁡k vs. the reciprocal of the cure temperature (1/T), as listed in Table 1; these findings are in coherence with Francucci et al. [41].

### 3.2. Gel Time and Vitrification Time Modeling

**Figure 4:**
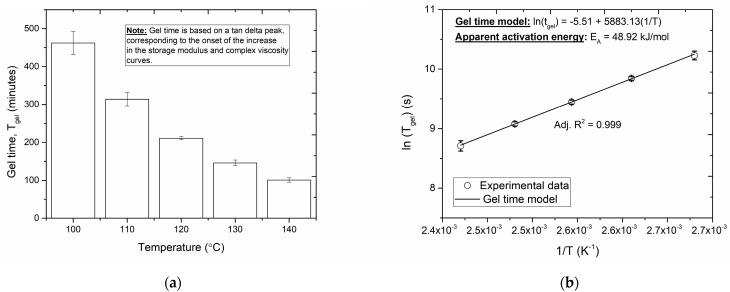
Gel time (**a**) as a function of isothermal cure temperature and (**b**) a comparison of the measured and predicted gel time with respect to the reciprocal of the isothermal cure temperature.

The gel times for the series of isothermal cure temperatures are summarized in Figure 4. The combination of the viscoelastic results with the cure conversion of each curing temperature allowed us to determine the degree of cure at the gel point ($\alpha_{gel})$. In the review works by Bilyeu et al. [42], the authors have summarized the various criteria that have been proposed in the literature to determine the gel point, such as the crossover point between the storage and loss moduli, the peak of the tangent delta, the onset of the increase in storage modulus, the peak in loss modulus, the inflection point of the storage modulus curve, and the onset of the plateau of the maximum value of the storage modulus. Therefore, in this study, the gelation time is estimated to occur at the onset of the increase in the storage modulus and complex viscosity curves.

**Figure 5:**
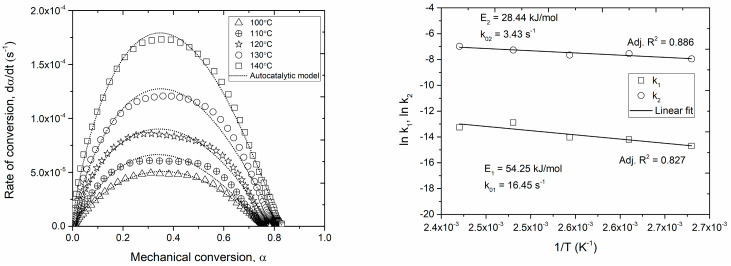
Rate of conversion as a function of the mechanical conversion for various isothermal curing (**left**), Arrhenius linear fit for the calculated kinetics constants (**right**).

A statistical average value of $\alpha_{gel}=$0.486 was obtained from the isothermal curing experiments, which appears to be comparably lower than that reported in the literature [43,44]. This may indeed be due to the aging of the prepreg. However, as the standard deviation of the data set is within the acceptable range (5.3%) to apply Flory’s gelation theory [33], Equation (6) was used to obtain a linear relationship between the gel time as a function of the cure temperature, as seen in Figure 4. The gradient of the straight line in Figure 5 is then used to obtain the apparent activation energy for the gel time model (48.92 kJ/mol).

### 3.3. T_g_-α Conversion

**Figure 6:**
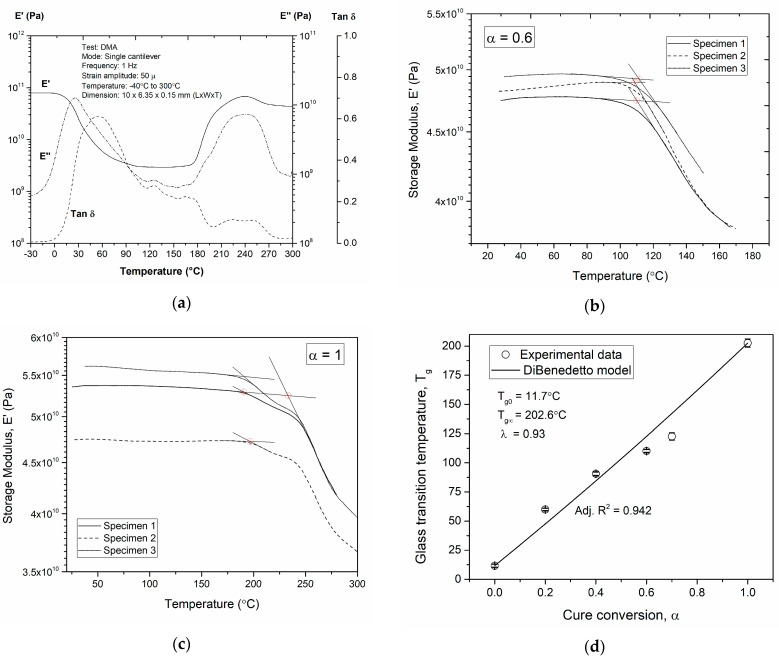
Glass transition temperature as a function of fractional conversion and relative DiBeneteddo modeling: (**a**) dynamic scan, (**b**) fractional degree of cure (0.6), (**c**) fully cured, (**d**) DiBeneteddo model.

The DiBenedetto model (Equation (7)) [35] is used to relate the glass transition temperature and the extent of reaction before gelation. The glass transition temperatures of the unreacted ($T_{g0}$) and the fully cured system ($T_{g\infty })$ were determined from a dynamic scan from −30 °C to 300 °C at a rate of 2 °C/min, while applying a strain amplitude of 50 µ at a frequency of 1 Hz. The experimental data to obtain the pre-defined degree of cure at $T_{g}$ was estimated by letting the towpregs cure at 120 °C for various periods of time such that they reached the required pre-defined degree of cure (estimations from the cure kinetics model), as seen in Figure 6b. Following this, the $T_{g}$ of all pre-cured samples were determined in a DMA as per ASTM D7028 [45] (Figure 6a). The wet glass transition temperature of the polymer, $T_{g0},$ and the fully cured $T_{g\infty }$ are determined in Figure 6, and the DiBenedetto model was used to predict the evolution of the $T_{g}$ as a function of cure conversion. In Figure 6d, the theoretical model provides a reasonable fit (94% match) with the experimental data of the $T_{g}$ evolution as a function α [46].

### 3.4. Manufacturing Cure Cycle

**Figure 7:**
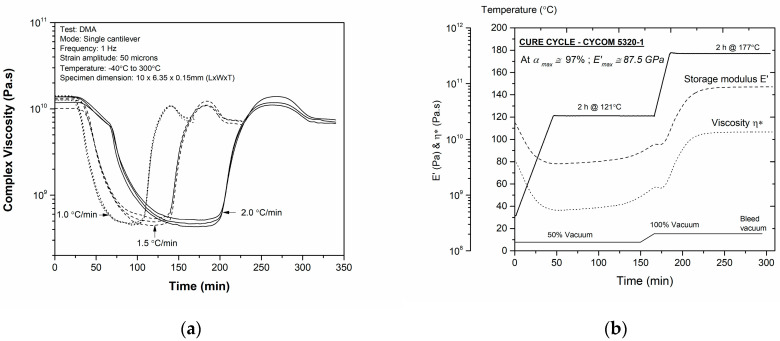
Cure cycle development for out-of-spec Cycom 5320-1: (**a**) complex viscosity curves used to determine the time taken to reach minimum viscosity, (**b**) temperature and pressure stages along with the achievable maximum cure.

The manufacturer’s recommended cure cycle for the vacuum bag only cure consists of several ramps and holds [47], which would invariably drive the cost of manufacturing. As sporting industry products are price sensitive compared to those in aerospace, it is important to keep the manufacturing costs to a minimum for product viability [48]. Therefore, in this work, a multi-step cure with two ramps is designed to cure the relatively thin laminates (<2 mm) using only a vacuum bag in a convection oven [49]. The two-step curing cycle with two ramps and two holds at 121 °C and 177 °C was designed based on the mechanical curing experiments using DMA techniques (Figure 7a). The multi-stage cure cycle (Figure 7b) consisted of a temperature ramp from ambient until approximately 120 (±5) °C at a rate of 2 °C/min, followed by a temperature isotherm dwell stage for approximately 1 h, during which edge devolatilization and temperature uniformity between the tooling and laminate is attained. The vacuum bag pressure was maintained at approximately 0.5 bar to prevent premature curing due to high vacuums. At the chosen temperature ramp rate, the resin attains its minimum viscosity at approximately 151 min, during which the vacuum pressure was increased to −1 bar to facilitate face bleeding (normal to the top laminate face) to remove any entrapped air and volatiles from the central areas of the laminate. The second stage of the temperature ramp was applied at the end of the temperature dwell until a cure temperature of 177 °C, where the actual curing of the resin takes place. At the end of the cure temperature dwell, the oven temperature is reduced gradually at approximately 2 °C/min until the laminate reaches at least 50 °C, following which the vacuum is bled [49].

### 3.5. Mechanical Tests, Extent of Cure, and Glass Transition T_g_

The mechanical test results show an approximately 13% and 15% decrease in tensile strength and modulus values, respectively (Table 2), compared to pristine material [47]. The reduction in tensile properties is analogous to the decrease in the cure properties and can be attributed to the maximum achievable cure of the out-of-spec material. However, the marginal change in properties may indeed not hinder their applicability in sports and recreational goods, as the structural requirements in those sectors are relatively lower compared to those in aerospace applications.

The extent of cure determined using the DSC methods on three cured samples exhibits an average cure of 95.4% (±1.2%) (Table 3), correlating well with the results of cure kinetics that were obtained using DMA methods (97% cure at 177 °C). While out-of-spec materials may not be suitable for the aerospace industry due to stringent rules and regulations around aged materials, it is still possible to use them in other practical applications, as they result in relatively good-quality laminates.

**Figure 8:**
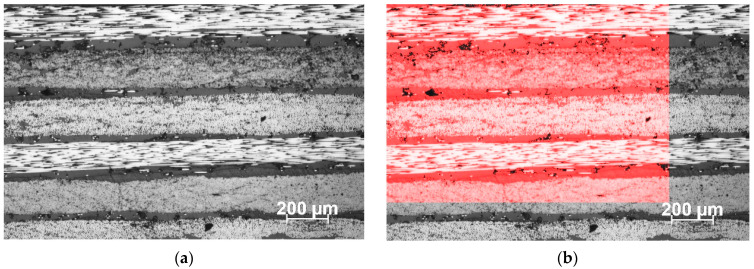
Optical micrograph of the cross-section of sample 1, with the red shaded region indicating ROI 1 that is used for void calculations.

The void fraction was determined using cross-sectional micrographs of three laminates (Figure 8). Three regions of interest (ROIs) of each sample were evaluated at 10× and 20× in Matlab^®^ (R2023a) environment, and the results are listed in Table 4. An example of one region that was considered for evaluation is shown in Figure 8a. The micrographs show an average of 2.4% in all the regions considered as seen in Figure 8b. This indicates that the out-of-spec material is still able to produce laminates with minimum voids compared to the non-expired material (0.15%) [50].

**Figure 9:**
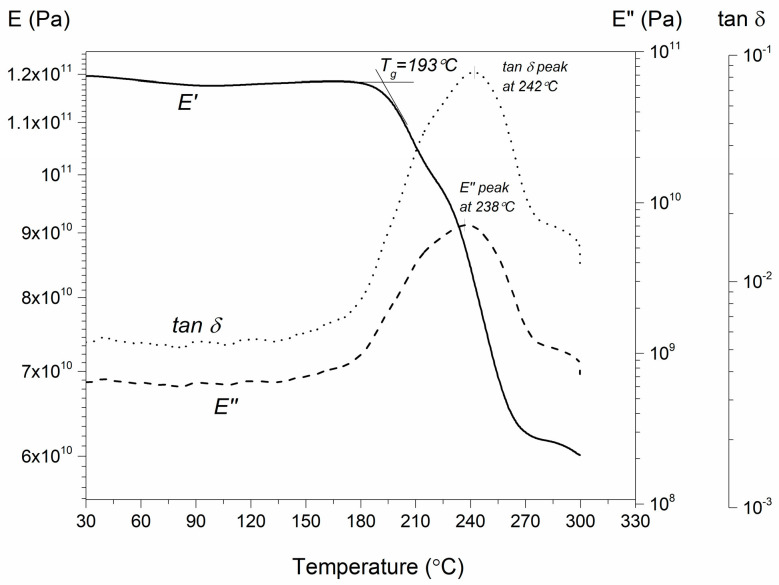
Mechanical properties’ time evolution during the DMA two-step cure cycle and evaluation of the T_g_ from the onset of the drop of the storage modulus.

The glass transition temperature (T_g_) of the cured laminate was determined in accordance with standard AITM-0003 [26] (Figure 9), considering the onset of the drop of the storage modulus (E′). The experimental T_g_ value (193.1 °C) is slightly lower compared to the one indicated by the manufacturer (196.7 °C). This expected result, on the one hand, proves that the maximum degree of cure and mechanical properties practically achievable for this prepreg cannot be obtained with an out-of-spec (expired) tape, and on the other hand, the quality of the laminate is not much lower. Therefore, while the material cannot be used for aerospace applications, it can be used for other purposes, such as sports equipment, where the necessary specifications are less strict.

## 4. Summary

In this study, cure kinetics of aged carbon prepregs were modeled using the mechanical cure conversion state of the material that was determined using a DMA machine. This method simulates the cure behavior of the entire fiber-resin system closely to the actual manufacturing process as opposed to the conventional DSC methods. The autocatalytic model, which was developed by comparing the storage modulus at the start of the reaction (E′_0_) and instantaneously (E′_t_) during the isotherm experiments with those of the fully cured material (E′_∞_) obtained from the dynamic scans, shows a reasonable match with experimental data. The maximum conversion at temperatures between 100 °C and 140 °C does not exceed 80% (±5%) and is found to be linearly related to the cure temperature. The aging of the prepreg has evidently lowered the degree of cure at the gel point to 0.486 compared to pristine ones. However, the evolution of the $T_{g}$ as a function of cure conversion that was modeled using the DiBenedetto equation provides a reasonable fit (94% match) with the experimental data, undermining the effect of viscoelastic results with the cure conversion $\alpha_{gel}$. The shorter multi-stage cure cycle that was developed results in shorter cycle times with negligible loss in the mechanical properties that can be attributed to the aging of the prepregs. The maximum cure that can be obtained in the laminates manufactured from the out-of-spec prepregs is 95.4%, which may indeed not be adequate for aerospace applications but is sufficient for other non-aerospace applications. 

Following the analysis, the out-of-spec material was used to manufacture tubing that was used to produce framesets for professional touring bicycles. The Mandel wrapping technique was used to manufacture the tubing that was cured in a convection oven using the designed cure cycle (Figure 7b). The bicycle was designed to withstand a 150 kg rider and handle severe crosswinds, and the weight of the bicycle frame is well under 1 kg. The product demonstrator developed showed that the aged prepregs can indeed be upcycled rather than being discarded as landfill.

## Figures and Tables

**Table 1:** Calculated kinetic parameters of the autocatalytic model.

Pre-Exponential Factors		Apparent Activation Energy		Reaction Order		Maximum Conversion	
$k_{01}$ (s^−1^)	16.45	$E_{a1}$ (kJ/mol)	54.25	m	0.86	$\alpha_{max}$	0.00172(T) + 0.119
$k_{02}$ (s^−1^)	3.43	$E_{a2}$ (kJ/mol)	28.44	n	1.11		

**Table 2:** Mechanical properties from the tensile tests conducted per standard ASTM C 3039.

	Tensile Strength (MPa)	Tensile Modulus (GPa)
Manufacturer’s data	917	59
Test coupons	800 (±20)	50 (±5)

**Table 3:** The extent of cure via DSC analysis conducted per standard AITM 3-0008.

Sample	∆H_100_ (J/g)	T_onset_ (°C)	T_peak_ (°C)	∆H (J/g)	α′ (%)
1	443.1	190.7	250.5	25.84	94.2
2	443.1	185.9	249.0	20.60	95.3
3	443.1	183.0	250.3	13.66	96.9

**Table 4:** Void content of manufactured samples at two magnifications.

		Resolution	
		10×	20×
Sample 1	ROI 1 (%)	2.5	1.9
	ROI 2 (%)	2.6	2.5
	ROI 3 (%)	2.3	2.8
Sample 2	ROI 1 (%)	2.8	2.4
	ROI 2 (%)	2.1	2.2
	ROI 3 (%)	2.2	1.8
Sample 3	ROI 1 (%)	2.5	2.6
	ROI 2 (%)	2.8	2.6
	ROI 3 (%)	1.8	2.8
Average		2.4	2.4
Standard deviation		0.3	0.3

## Data Availability

Data are contained within the article.
